# EARLY PARENT–CHILD RELATIONSHIPS, BURDEN, AND SUPPORT AMONG ADULT CHILDREN OF PARENTS WITH DEMENTIA

**DOI:** 10.1093/geroni/igae098.2743

**Published:** 2024-12-31

**Authors:** Kristie Wood, Denise Chow, Thi Hoang Vu, Joan Monin

**Affiliations:** Colorado University Anschutz School of Medicine, Aurora, Colorado, United States; Yale University, New Haven, Connecticut, United States; Yale University School of Public Health, New Haven, Connecticut, United States; Yale School of Public Health, New Haven, Connecticut, United States

## Abstract

Research has demonstrated that greater childhood adversity is associated with poor mental health in older adult family caregivers. Yet, it is unknown whether early parent-child relationship quality is more specifically associated with adult children’s caregiver burden and perceived support from their parents living with cognitive impairment. We examined associations between early life parent-child relationship quality, current caregiver burden, and adult children’s perceived support from their parents, among adult children of parents living with early-stage dementia. Adult children of parents living with cognitive impairment (n=144, m=47.53, sd=11.2, Non-Hispanic=92%, White=72%) completed the following self-report measures: Parental Bonding Inventory, Zarit Burden Interview, and Perceived Partner Responsiveness Scale. Multiple linear regression models revealed children who identified more care, less overprotection, and optimal parenting in their early parent-child relationships reported greater current perceived support from their parents. However, there was no statistically significant association found between early parent-child relationship quality and caregiver burden. Our findings indicate that while early positive relationship quality may be indicative of adult children’s future perceptions of support from their parents with early-stage dementia, it may not buffer against perceptions of caregiver burden. Overall, results highlight that early parent-child relationship quality may have consequences for caregiving relationships in later life. Future research is needed to explore mechanisms of early parent-child relationship quality and perceptions of support that are associated with caregiver outcomes.

